# The Appearance of Unknown Diseases

**Published:** 1881-10

**Authors:** 


The Appearance of Unknown Diseases.—It cannot fail to strike a careful reader of the newspapers, that there is a very large number of very strange diseases constantly appearing among the cattle or horses in different parts of the country. In the past few months we have noticed announcements of such troubles in a half a dozen different quarters. There has been a "mysterious disease" attacking the cattle or horses in New Jersey, New York, Nova Scotia, Illinois, Colorado, Nebraska, Oregon and other places. The story is almost a stereotyped one : "Veterinarians are unable to give a name to it." These facts do not speak well for the local veterinarians, since in most cases the disease is not so mysterious after all. It is generally some form of anthrax, or perhaps some bowel or lung complaint, or an influenza.

We beg our veterinary readers hereafter to furnish a diagnosis, of some kind at least, to the associated press. It does not look well to have it go abroad that our cattle are subject to four or five new kinds of diseases every year.

We should be very glad to receive accounts of outbreaks of these pathological mysteries from the local veterinarians, whenever such occur.

